# Gut microbiota: a hidden player in polycystic ovary syndrome

**DOI:** 10.1186/s12967-025-06315-7

**Published:** 2025-04-15

**Authors:** Harshini Senthilkumar, Mohanapriya Arumugam

**Affiliations:** Department of Biotechnology, School of Biosciences and Technology, Vellore Institute of Technology, Vellore, Tamil Nadu 632014 India

**Keywords:** Dysbiosis, Gut microbiota, Hormonal metabolism, LPS, PCOS, SCFA

## Abstract

Polycystic ovary syndrome (PCOS) is an endocrine disorder that affects reproductive-aged women worldwide, causing hormonal imbalances and ovarian dysfunction. PCOS affects metabolic health and increases the risk of obesity, insulin resistance, and cardiovascular disease, in addition to infertility. This review delves deeper into the connections of gut microbiota with PCOS pathophysiology, particularly into its impact on hormone metabolism, obesity, inflammation, and insulin resistance by way of short-chain fatty acids, lipopolysaccharides, and gut-brain axis. Studies also show that changes in the metabolic processes and immune responses are seen in changes in the gut microbiota in PCOS subjects, such as changes in the *Bacteroidetes* and *Firmicutes* groups. Some bacteria, like *Escherichia* and *Shigella*, have been associated with dysbiosis in patients with PCOS, leading to systemic inflammation and changed hormone levels, which further worsen the clinical symptoms. Therapeutic interventions targeting the gut microbiota comprise probiotics, prebiotics, and fecal microbiota transplantation; these have potential to alleviate the symptoms of PCOS. Other precision microbiome-based therapies include postbiotics, and CRISPR-Cas9 genome editing, which are relatively new avenues toward precision treatment. This complex interlink of gut microbiota and PCOS pathophysiology will open the avenues for possible treatments for hormonal imbalances and metabolic problems that characterize these complex disorders. The review here focuses on the requirement of further studies to be able to elucidate the specific pathways relating gut microbiota dysregulation to PCOS and, thus, improve microbiome-based therapies for better clinical outcomes in affected individuals.

## Graphical Abstract

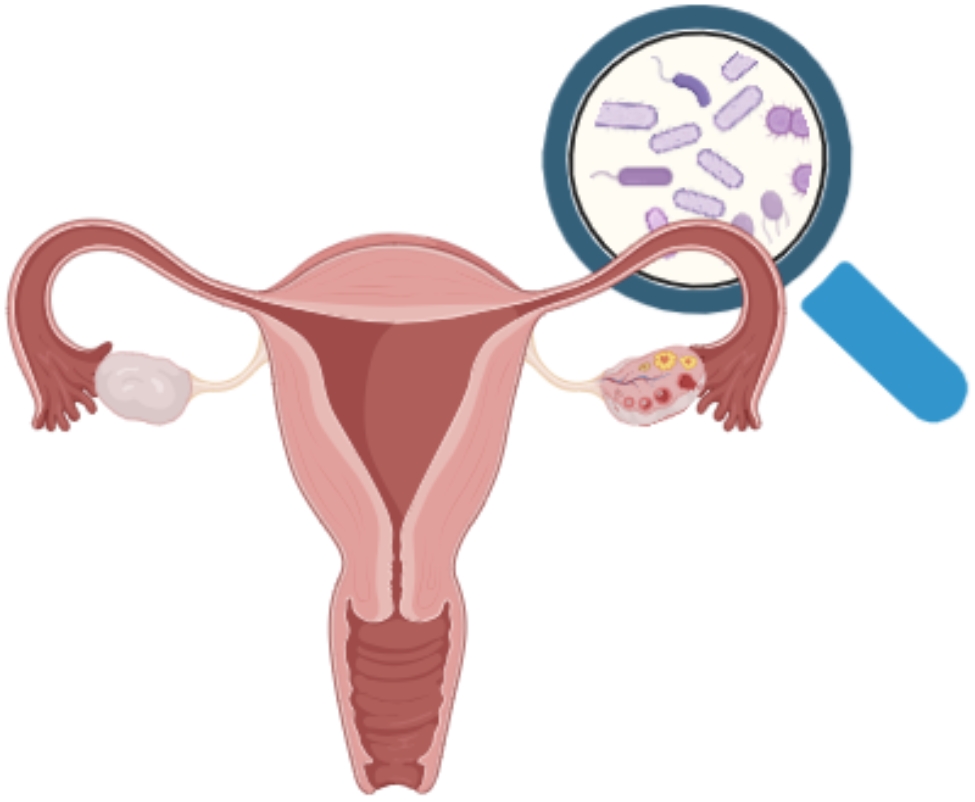

## Introduction

Polycystic ovarian syndrome, or PCOS, is the most prevalent cause of endocrine infertility in women. It is a complicated and diverse disease characterized by anovulation, increased ovarian androgen production, and infertility [1, 2]. Despite this, PCOS has a wide-ranging impact on women’s health, with long-term consequences that extend far beyond the reproductive years [3, 4]. According to a thorough screening procedure that used the National Institutes of Health (NIH) diagnosis standards, 4–10% of women in the world are thought to have PCOS [5]. The National Institutes of Health sponsored the Evidence-Based Methodology PCOS Workshop in 2012, which classified PCOS into four phenotypes: phenotype A (hyperandrogenism, polycystic ovary morphology, and ovulatory dysfunction), phenotype B (excess testosterone and ovulatory dysfunction), phenotype C (excess testosterone and polycystic ovary morphological characteristics), and phenotype D (ovulatory dysfunction and polycystic ovary morphological characteristics) [6, 7]. The actual diagnosis criteria are based on three criteria: the National Institutes of Health Consensus (2012), the Androgen Excess and PCOS Society (2006), and the Rotterdam 2003.

The most comprehensive and often applied of these criteria is the Rotterdam criterion [8]. These criteria offer three characteristics: (1) oligo-anovulation, (2) clinical or biochemical hyperandrogenism, or both, and (3) polycystic ovary morphology (PCOM), which is defined by ultrasound sonography showing the existence of 12 or more follicles with a maximum diameter of 2 to 9 mm or any ovarian volume greater than 10 mL [9]. Aside from its influence on fertility, polycystic ovarian syndrome can lead to long-term complications. Previous research has demonstrated that PCOS is commonly associated with metabolic disorders, including obesity, insulin resistance (IR), and cardiovascular disease [10].

PCOS has been linked to metabolic changes that may involve alterations in the gut microbiota. The gut microbiota carries a vast quantity of information. It is generally known that the amount of bacteria in the body is about equal to the number of human cells [11], and that the quantity of genetic information present in these microorganisms is at least 150-fold more than that in the human genome [12]. Hormones secreted by commensal bacteria can influence host metabolism, immunity, and behaviour through interactions with other microorganisms. Every phase and degree of female reproduction is impacted by the human microbiome, including ovarian follicle and egg development, fertilization and embryo migration, implantation of eggs, and all stages of pregnancy, including parturition [13].

Alterations in the microbiome, particularly the gut microbiome, may influence the reproductive endocrine system, and rectifying abnormal microbiomes could result in better reproductive results [14]. Several studies have identified specific linear relationships between gut microbiota and serum hormone levels, which may have an additional effect on the general state of the body [13]. According to studies, the gut microbiota of PCOS patients may be associated with the development and occurrence of hyperandrogenism, insulin resistance, chronic inflammation, and metabolic syndrome, and may influence the clinical manifestations of PCOS via short-chain fatty acids, sex hormones, lipopolysaccharides and the brain-gut axis [15, 16].

## Microbiota composition and diversity in PCOS

**Fig. 1:**
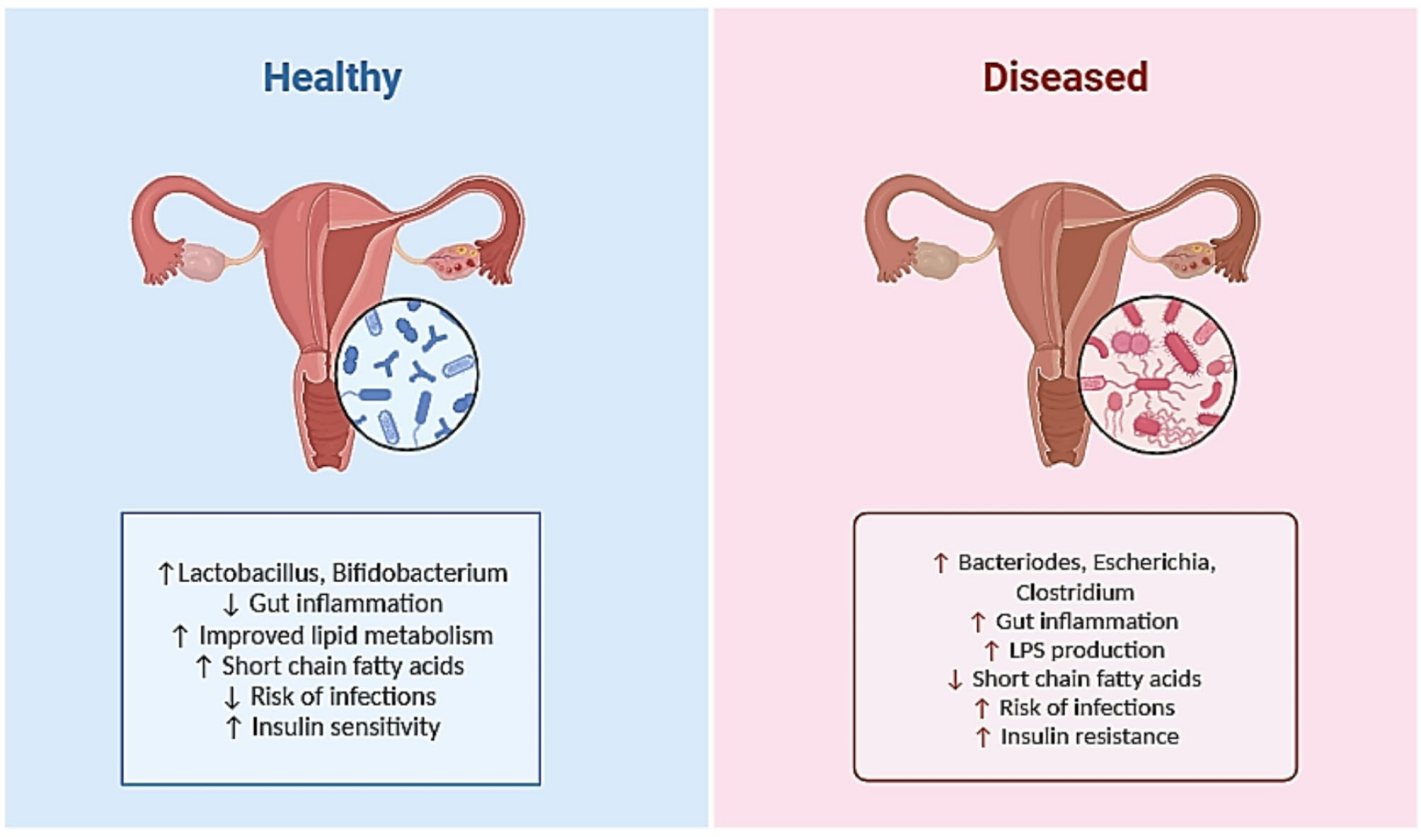
Illustrates gut microbiota and metabolic outcomes are compared in healthy (left) and diseased (right) states, highlighting differences in inflammation, bacterial composition, lipid metabolism, short-chain fatty acids, infection risk, and insulin sensitivity

The human gut microbiome comprises around 10^13^ to 10^14^ bacteria, over 1000 distinctive species, and more than 7000 strains [11, 17]. In the gut microbiome, bacteria predominate, especially anaerobes, while viruses, protozoa, archaea, and fungi are also present [11]. The two most common taxa of bacteria are *Firmicutes* and *Bacteroidetes*, whereas *Proteobacteria*, *Actinobacteria*, *Fusobacteria*, and *Verrucomicrobia* are found in substantially fewer numbers [18]. In addition to an alteration in the overall composition of the microbiome, studies have shown that PCOS causes an alteration in the balance of some bacteria species, such as *Bacteroidetes* and *Firmicutes* [19–21], the alteration can lead to altered production of short-chain fatty acids, which has an adverse effect on metabolism, gut barrier integrity, and immunity [22]. Figure 1 illustrates these changes by comparing the effects of gut bacteria in healthy and diseased states.

Modifications in the alpha and beta diversity in the gut microbiota have been linked by a few studies to PCOS. Some research suggests that PCOS is associated with reduced alpha diversity and altered beta diversity, whereas others show that microbial diversity remains intact [23]. Instead, Qi et al. observed an increase in *Bacteroides vulgatus* abundance, accompanied by a decrease in glycodeoxycholic and tauroursodeoxycholic acid levels, which leads to a shift in IL-22 levels [24]. In terms of *Bacteroides* genera, Liu et al. found a particular increase of *Escherichia* and *Shigella* in PCOS women, as well as a general gut microbiome composition comparable to obese control women [19]. The intestinal microbiome dysbiosis of obese and PCOS women was identical. Obese PCOS women revealed a more severe gut microbiota dysbiosis than those with simply PCOS or obesity. Gram-negative bacteria from the *Escherichia*/ *Shigella* and *Bacteroides* genera were found in considerably greater quantities in the intestines of obese PCOS women. Bacterial lipopolysaccharides [LPS] generated by gram-negative bacteria was demonstrated to cause chronic inflammation, obesity, and insulin resistance in LPS-infused rats [19]. Sexual hormones, including testosterone, estradiol, and progesterone, are involved in a variety of physiological processes, including inflammation, metabolism, differentiation, cell division, homeostasis, apoptosis and brain function. These hormones also play a part in communication between microorganisms and their hosts [25]. Antibiotic usage is associated with reduced estrogen levels, indicating that intestinal bacteria play an essential role in estrogen metabolism [26]. Also, estrogen levels can influence the status of illnesses and processes such as PCOS, endometrial hyperplasia, and eventually, fertility [27]. Beneficial bacteria, such as *Lactobacilli* and *Bifidobacteria*, which improve immunity and absorption of nutrients, are considerably decreased in PCOS patients [28, 29]. The gut microbiome changes in PCOS are distinctive, often contentious, and not entirely understood. However, some research has attempted to study the link between intestinal microbiota and PCOS; the majority of them have focused on the interaction between gut bacteria with insulin resistance, obesity and sex hormones.

## Dysbiosis of gut microbiota in PCOS

**Fig. 2:**
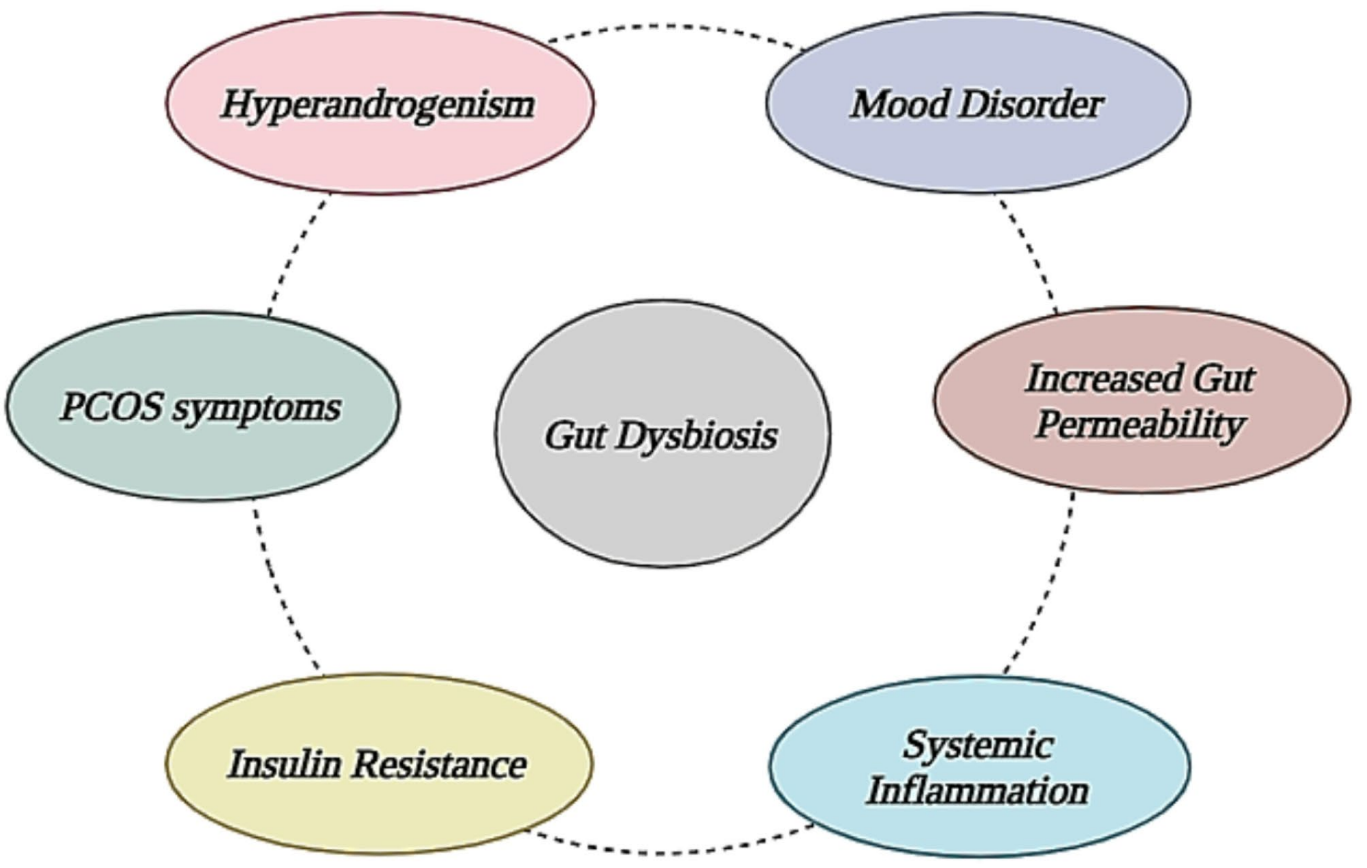
Illustrates the effects of gut dysbiosis in the development of PCOS. Gut dysbiosis leads to increased gut permeability, causing the translocation of microbial compounds to enter systemic circulation. It leads to chronic systemic inflammation that contributes to insulin resistance, hyperandrogenism, and mood disorder, all characteristics of PCOS. These metabolic and hormonal disorders emerge clinically as irregular menstrual periods and hirsutism

The gut microbiota serves as the “endocrine organ” for regulating and sustaining human health. Gut microbiota impacts the reproductive endocrine system via interacting with estrogen, androgen, insulin, and so on [12]. Gut microbiota dysfunction **is** linked to PCOS symptoms, including hyperandrogenism, insulin resistance, chronic inflammation, and aberrant brain gut peptide levels [30]. As a result, gut microbiota may influence follicular growth, sex hormones, and metabolic levels via hyperandrogenism, insulin resistance, chronic inflammation, the brain-gut axis, and other mechanisms, that influence PCOS pathogenesis. There are several possible mechanisms that explain the role of the gut microbiota in the pathogenesis of PCOS. The potential factors linking gut dysbiosis to the development of PCOS are shown in Fig. 2. *Lactobacilli* and *bifidobacteria* constitute beneficial bacteria of the family that improve immunity and nutrition absorption. Patients with PCOS showed a significant decrease in these microorganisms. Zeng et al. found that PCOS patients had substantially fewer *Prevotellaceae* than healthy people. *Prevotellaceae* levels have been shown to be more widespread in rodent models, indicating an imminent inflammatory response. Torres et al. discovered four genus-level taxa (*Anaerococcus*, *Roseburia*, *Odoribacter* and *Ruminococcus*) with reduced abundances in PCOS patients [31].

Conversely, metagenomic species analysis in certain studies showed that various bacteria, including *Shigella*, *Bacteroides fragilis*, *Parabacteroides merdae*, and *Escherichia*, are enriched. Further analysis of a few common strains led them to suggest some possible mechanisms linking dysbiosis of the microbiome and PCOS: microbiota may damage intestinal gut permeability and induce intestinal tract barrier dysfunction; certain bacteria may produce large amounts of reactive oxygen species; and enriched Gram-negative bacteria may produce LPS [32]. Gram-negative bacteria create LPS, which penetrates the “leaky gut” wall and enters the bloodstream, causing chronic low-grade inflammation. Following intestinal mucosal damage, LPS enters the bloodstream and causes endotoxemia. TLR4 recognizes and binds LPS via the LPS binding protein (LBP), CD14, and bone marrow differentiation factor-2 (MD-2) [33]. Inflammatory mediators and cytokines may be expressed in response to LPS. The inflammatory response can be triggered by the expression of inflammatory substances such as interleukin 6 (IL-6). Remarkably, IL-6 has been demonstrated to enhance the expression of various enzymes responsible for androgen synthesis, of which CYP17A1 is an important enzyme in the biosynthesis of androgens, such as testosterone. This inflammatory chain is thus connected to elevated testosterone production by the ovaries [34]. Insulin resistance appears to be the primary cause of metabolic problems in PCOS patients, promoting a chronic inflammatory state [35]. Immune system activity interacts with insulin receptors, raising insulin levels and increasing testosterone synthesis in the ovary, resulting in PCOS. The dysbiosis of gut microbiota, explained by the DOGMA hypothesis, provides a potential framework for understanding the gut’s role in PCOS pathophysiology [36].

The gut microbiome may potentially have an essential role in the progression of Insulin Resistance and the mechanisms linking IR, gut microbiota, and PCOS. Zeng et al. discovered that PCOS patients with insulin resistance exhibited decreased levels of *Prevotella* and greater levels of *Bacteroides* species in their intestinal microbiota compared to the healthy control group. Gut microbiota may impact bile acid metabolism and cause insulin resistance [37]. The metabolism of bile acids and insulin resistance may be impacted by gut microbiota. Studies indicate that PCOS patients have an enormous rise in common *Bacteroides* in their intestinal bacteria, perhaps due to reduced IL-22, insulin resistance, and bile acid production [24]. Microbial enzymes, such as BSH or bile salt hydrolase, hydrolyse primary bile acids to secondary bile acids, and these interact with receptors such as farnesoid X receptor (FXR), and G-protein coupled bile acid receptor (TGR5). Stimulation of these receptors impacts metabolism and hormonal processes, including those within the hypothalamic-pituitary-ovarian (HPO) axis controlling ovarian androgen production, further increasing testosterone [38].

Qi et al. performed a fecal microbiota transplant (FMT) from normal-weight PCOS women into antibiotic-treated mice to discover reproductive and metabolic changes in the recipients. Trans-PCOS mice (FMT from women with PCOS) had noticeably greater amounts of testosterone and luteinizing hormone (LH) than trans-control mice (FMT from healthy persons) [24]. The decrease in gut microbiota diversity and dysbiosis leads to a reduction in β-glucuronidase activity, which causes a decrease in the conversion of estrogen and phytoestrogen into its active and circulating forms. The reduction in circulating estrogens affects estrogen receptor activation, potentially leading to hypoestrogenic diseases such as metabolic syndrome, cardiovascular disease, obesity and cognitive decline [13, 39].

Tremellen and Pearce hypothesize that gut dysbiosis, especially impacted by a high-carbohydrate diet and a high-fat diet (HFD), causes inflammation through gut barrier disruption, which leads to IR, HA, and ovarian dysfunction [35]. Alpha [19, 20, 40] and beta diversity [20] were correlated with HA, demonstrating that elevated testosterone levels are associated with alterations in the general composition of the gut microbial population. According to these findings, people with PCOS have a different gut microbiota diversity and quantity of associated bacteria. Patients with PCOS may have abnormal intestinal mucosa integrity and altered metabolism as a result of an inflammatory response triggered by their intestinal microbes [41].

A study by Jobira et al., 2020, observed significantly lower α-diversity among obese PCOS patients compared with obese controls; thus, suggesting that there were reduced microbial richness and evenness. Moreover, the β-diversity analysis indicated different microbial communities clustering among obese PCOS patients and controls, which would mean that significant compositional differences existed. Notably, *Prevotella* and *Bacteroides* species levels, associated with inflammation and insulin resistance, respectively, were grossly elevated in obese PCOS whereas beneficial microbes including *Akkermansia* and *Ruminococcaceae*, which mediate gut barrier integrity and anti-inflammatory function, were strongly reduced [40]. Liu et al. (2017) have studied the gut microbiota composition in obese and non-obese women with and without PCOS, showing a significant imbalance in the microbial community of PCOS patients. The results showed that gram-negative bacteria, such as *Bacteroides* and *Escherichia*/*Shigella*, were more abundant in women with PCOS, especially in obese women. These bacteria produce LPS, which is linked to chronic inflammation, insulin resistance, and obesity. Further, it also observed the decrease of beneficial bacteria like *Akkermansia* that are involved in the prevention of metabolic endotoxemia and maintaining gut barrier. Gut microbial imbalance is pronouncedly more severe among obese women as compared to non-obese women indicating that the effects of obesity are combined with PCOS on altering the gut microbiota. These changes in gut microbiota correlated with increased BMI and testosterone levels, suggesting their possible contribution to the pathophysiology of PCOS through inflammatory and metabolic pathways [19].

## Mechanisms linking gut microbiota dysregulation to PCOS

A study conducted in 2016 observed that patients with insulin resistance exhibit a greater quantity of branched-chain amino acids (BCAAs) in their serum metabolome. These amino acids are associated with a higher number of species, including *Prevotella copri* and *Bacteroides vulgatus*. Also, *P*. *copri* may exacerbate glucose intolerance, elevate BCAA levels, and provoke insulin resistance [42]. Further supporting the results of the 2016 study, Gojda & Cahova (2021) reaffirmed the role of *Prevotella copri* and *Bacteroides vulgatus* in altered BCAA metabolism and insulin resistance. This study showed that *P. copri* contributes to increased BCAA levels and glucose intolerance, reinforcing the association between gut microbiota and metabolic disorders [43].

Gastrointestinal hormones such as Ghrelin and peptide YY (PYY), which both have a negative connection with IR and BMI, might represent another potential relationship between the gut microbiota and IR observations [20, 44]. Liu et al. found that women with PCOS have lower levels of ghrelin and PYY than healthy women, perhaps due to an increase in *Bacteroides* species that are adversely linked with ghrelin [20]. The brain-gut axis is a biphasic signaling system. Numerous mechanisms exist for the microbiome to impact the brain-gut axis. The brain can receive messages from the gut bacteria by stimulating the vagus nerve pathway. Complex information in the digestive tract can be conveyed to the brain via synapses generated by the myenteric plexus of efferent nerve terminals and postganglionic neurons. By producing hormones and neurotransmitters, the gut microbiome simultaneously provides feedback to the brain [45]. Recent research suggests that PCOS may affect not just the hypothalamic-pituitary-ovarian axis, but also the gut-brain axis. This pathway is connected by intricate two-way communication mechanisms in the brain-gut axis such as central nervous system and gastrointestinal system. Research indicates that gut-brain axis mediators including serotonin, ghrelin, and PYY) can regulate appetite, metabolic rate, and LH production. According to reports, these mediators may help women with PCOS maintain psychological well-being and control gastrointestinal habits [20, 46].

**Fig. 3:**
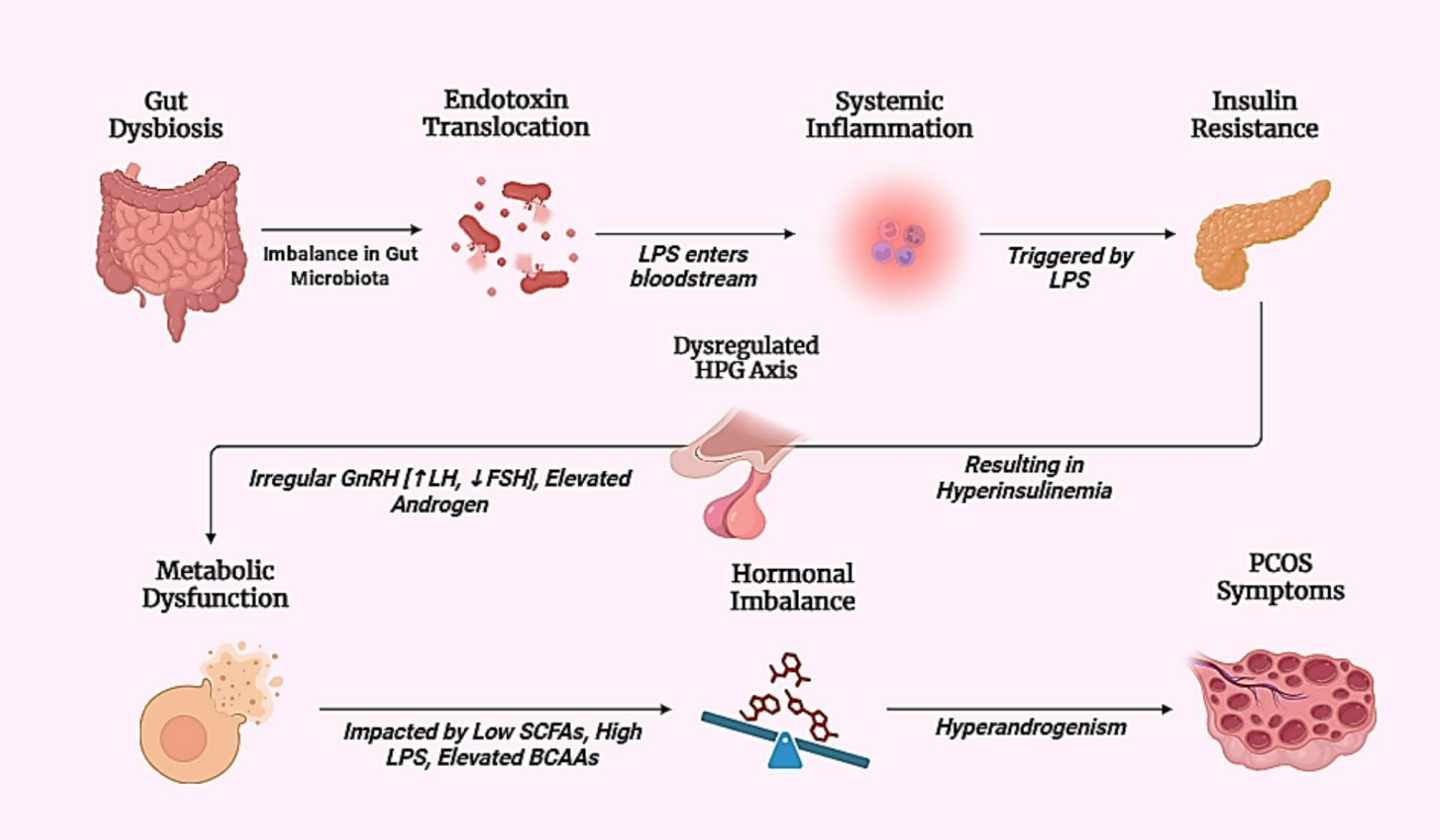
Illustrates that gut dysbiosis results in LPS translocation into the blood and leads to systemic inflammation, causing insulin resistance and hyperinsulinemia. The inflammation is linked to disturbances of the HPG axis due to disruption in GnRH secretion, an imbalance in LH levels to FSH, and overproduction of androgens. Besides this, imbalanced SCFAs and elevated BCAAs also promote hormonal imbalance through metabolic dysfunction and enhance hyperandrogenism. These disruptions become symptoms of PCOS, like irregular menstrual cycles, infertility, hirsutism, and acne

Previous studies have established that alterations of the gut microbiota produced by a poor diet lead to greater gut permeability and the subsequent transfer of LPS from Gram-negative bacterial colonies into systemic circulation [36]. Figure 3 shows the pathway from gut dysbiosis to PCOS symptoms, illustrating gut microbiota disruptions that lead to hormonal imbalances, insulin resistance, chronic inflammation and hyperandrogenism. The increased Bacteroides population caused by elevated LPS interacts with bile acids, resulting in abnormal metabolism of lipids in PCOS [47]. Chronic endotoxemia is caused by excessive gram-negative bacteria growth in obese people, which increases the amount of systemic bacterial LPS. Bacterial lipopolysaccharide is recognized by toll-like receptors (TLR) on the surface of enterocytes, which then trigger the nuclear factor kappa B (NF-kB) pathway to cause inflammation. Endotoxemia is also caused by a shift in gut microbiota, a reduction in tight junction protein expression, and an increase in intestinal mucosal permeability. High-fat diet (HFD) are known to raise intestinal permeability and LPS translocation [20, 48]. To investigate the relationship between Bacteroides and bile acids, Qi et al. [24] conducted an investigation in mice and discovered that bile acids such as tauroursodeoxycholic acid (TUDCA) and glycodeoxycholic acid (GDCA) decrease in PCOS while Bacteroides vulgatus populations increase. This results in ovarian dysfunction and insulin resistance. A reduction in IL-22 levels impacts ovarian shape and follicle development whereas GDCA promotes the PCOS phenotype by binding to ILC3 (innate lymphoid cell type 3) and GATA3 (GATA binding protein 3) and raising IL-22 production [24]. This result was confirmed when low levels of IL-22 were discovered in PCOS patients [47].

The pathophysiology of PCOS involves hyperandrogenism, which is a significant factor [49]. Common clinical symptoms include hirsutism and acne. Research indicates that hyperandrogenism is linked to gut microbiota [50]. Testosterone may impact the gut microbiota composition in females. A regression analysis revealed that reduced abundances of certain genera were associated with elevated circulating testosterone concentrations and deprived metabolism of glucose in PCOS mice [21]. Using a testosterone cypionate-induced mouse method, the fecal microbiome of pre-natal androgen (PNA) mice had an elevated relative abundance of bacteria involved with steroid hormone synthesis and short-chain fatty acid (SCFAs) metabolite formation [28]. In laboratory studies of PNA exposure and maternal HA, the relative number of bacteria involved with steroid production (*Nocardiaceae* and *Clostridiaceae*) and propagation of unsaturated SCFAs increases while *Akkermansia*, *Bacteroides*, *Lactobacillus*, and *Clostridium* decreases. These mice additionally exhibited increased body weight and adipokine mRNA expression, and cardiovascular function was impaired due to elevated systolic and diastolic blood pressure and a reduced heart rate [28]. The human gut microbe *Clostridium scindens*, possesses genome contains 20α-hydroxysteroid dehydrogenase (HSDH), has a high potential to convert glucocorticoids into androgens [51]. The primary metabolites of indigestible carbohydrates (like dietary fiber) generated by bacteria are called SCFAs, and they play a significant part in the prevention as well as the treatment of obesity [52, 53]. SCFAs modulate systemic metabolism and hormonal pathways through the activation of G-protein-coupled receptors (GPR41 and GPR43) and inhibition of histone deacetylases (HDACs) [54]. Through G protein-coupled receptors (GPR41 and GPR43), SCFAs induce enteroendocrine cells to release glucagon-like peptide 1 (GLP-1) and peptide YY (PYY) [55]. These mechanisms have a significant impact on inflammation, insulin resistance, and ovarian steroidogenesis. SCFAs increase insulin sensitivity by promoting GLP-1 and PYY, which cause hyperinsulinemia, a well-known stimulator of androgen synthesis in ovarian theca cells. Hyperinsulinemia increases the expression of steroidogenic enzymes, such as cytochrome P450c17 (CYP17A1), in theca cells. This leads to increased synthesis of androgens like testosterone [56]. Through a PPARγ-dependent transition from lipogenesis to fat oxidation, SCFAs have positive metabolic impacts on protection against obesity caused by an HFD [33]. Table 1 describes gut microbiota alterations in PCOS patients from various studies utilizing 16 S RNA sequencing techniques.

**Table 1:** Summary of studies investigating gut microbiota composition in PCOS

Author	Country	Sample Characteristics	Technique	Findings	Reference
He, F., & Li, Y. (2021)	China	PCOS-IR (*n* = 14), PCOS-NIR (*n* = 12), Control (*n* = 10)	16 S RNA sequence V3-V4	↑ *Enterococcus*, *Rothia*, *Ruminococcus* ↓ *Prevotella*	[48]
Fangyuan et al., (2019)	China	PCOS (*n* = 10), MSA-PCOS (*n* = 10), Control (*n* = 10)	16 S RNA sequence V3-V4	↑ *Bacteroides* ↓ *Prevotella*, *Faecallibacterium*	[48]
Liu et al., (2017)	China	PCOS non-obese (*n* = 12), PCOS obese (*n* = 21), Control non-obese (*n* = 9), Control obese (*n* = 6)	16 S RNA sequence V3-V4	↑ *Bacteroides*, *Escherichia*/*Shigella* & *Streptococcus* ↓ *Akkermansia* & *Ruminococcaceae*	[19]
Zeng et al., (2019)	China	PCOS-IR (*n* = 9), PCOS-NIR (*n* = 8), Control (*n* = 8)	16 S RNA sequence V3-V4	↑ *Bacteroidaceae* ↓ *Prevotellaceae*	[41]
Lindheim et al., (2017)	Austria	PCOS (*n* = 24), Control (*n* = 19)	16 S RNA sequence V1-V2	↓ Phylum *Tenericutes* (ML615J-28), Phylum *Bacteroides* (S24-7)	[57]
Mammadova et al., (2021)	Turkey	PCOS lean (n-22), Control (*n* = 24)	16 S RNA sequence V3-V4	↑ *Clostridium* cluster XVII, ↓ *Clostridium sensustricto* & *Roseburia*	[58]
Dong, S., et al. (2021)	China	PCOS (*n* = 45). Control (*n* = 37)	16 S RNA sequencing	↑ *Ruminococcus*, *Prevotella*, *Bacteroides* ↓ *Firmicutes*	[59]
Yu, Z., et al. (2022)	China	PCOS (*n* = 20), Control (*n* = 20)	16 S RNA sequencing V3-V4	↑ *Escherichia*, *Shigella*, *Megamonas*	[60]

## Therapeutic approaches targeting gut microbiota in PCOS

The interaction between the microbiome and PCOS pathogenesis has been better characterized, and a link between diet, microbiota, and PCOS may be suggested, showing the potential influence on PCOS prevention and treatment. Research has demonstrated the distinct effects of plant-based and animal-based proteins on the gut microbiota. Specifically, it has been observed that plant-based proteins decrease the numbers of *Bifidobacterium* (B.) *adolescentis* and increases *Bacteroides*, *Alistipes*, and *Bilophila*, hence elevating the risk of cardiovascular disease. On the other hand, plant proteins have been shown to boost *Bifidobacterium* and Lactobacillus while lowering *Bacteroides fragilis* and *Clostridium perfringens*, resulting in increased SCFA levels and decreased inflammation [61, 62]. Table 2 outlines various therapeutic interventions aimed at modulating gut microbiota in patients with PCOS.

**Table 2:** Therapeutic interventions against gut microbiota in PCOS

S.No	Treatment	Mechanism	Examples	References
1.	Probiotics	Increase in SCFA production, Prevent pathogenic bacteria, Reduce serum insulin concentration, Decrease luminal pH	*Lactobacilli*, *Bifidobacterium*	[63]
2.	Prebiotics	Improve blood lipid profile and glucose, Reduce serum Triglycerides, Reduce fasting plasma glucose and LDL cholesterol	FOS, GOS, Inulin	[64]
3.	Synbiotics	Anti-inflammatory, Produce SCFAs, Improve insulin sensitivity, Decrease atestosterone level	*Lactobacillus* + *Lactitol* *Bifidobacterium* + FOS *Bifidobacterium* + GOS	[65]
4.	Psychobiotics	Anti-depressants, Prevent inflammation, Reduce cortisol levels, Treats anxiety, depression and mood disorder	*Lactobacillus plantarum*, *Bacillus infantis*, *Lactobacillus helveticus*	[31, 32]
5.	Dietary changes	Anti-inflammatory effect, SCFA production, Improve metabolic and hormonal profiles	High-fiber diet, Low-sugar diet	[65]

FMT is an updated therapy for inflammatory bowel disease. FMT involves transplanting healthy feces into patient’s digestive tracts to improve and rebuild the gut microbiota and treat disorders [58]. A research found that implementing an FMT from healthy rats into a rat model of PCOS generated by letrozole resulted in a drop in androgen levels, enhanced estrous cycles, normalized ovarian morphology, and a decrease in *Prevotella* and an increase in *Lactobacillus* and *Clostridium* species. An in vivo study found that FMTs raise estrogen levels, lower blood androgen levels, and support a normal menstrual cycle [29].

The World Health Organization (WHO) defines probiotics as “live micro-organisms when administered in sufficient quantities, promote beneficial health effects on the host” [66]. Naturally occurring probiotic bacteria may be found in fermented foods. They have anti-inflammatory, anti-microbial, and anti-oxygenic properties as well as improve metabolic parameters, alter the gut microbiota, and control the immune system. Probiotic supplementation has shown promising results in improving metabolic and hormonal parameters in women with PCOS. In a 12-week randomized controlled trial, supplementation with probiotics containing *Lactobacillus* and *Bifidobacterium* strains resulted in significant reductions in body weight, body mass index (BMI), fasting plasma glucose, insulin levels, and Homeostatic Model Assessment of Insulin Resistance (HOMA-IR). In addition, beneficial effects on lipid profiles were observed, including reductions in Triglycerides (TG) and Very Low Density Lipoprotein (VLDL) cholesterol levels [62, 63]. Elevated TG and VLDL levels are important markers of metabolic disorders in women with PCOS. Insulin resistance, a common feature in PCOS, leads to increased liver production of VLDL, leading to hypertriglyceridemia. This lipid imbalance contributes to atherogenic changes and increases the risk of cardiovascular disease in PCOS patients [67, 68]. Women with PCOS experienced identical outcomes after 8 weeks of consuming *L. rhamnosus*, *L. casei*, *L. acidophilus*,* L. bulgaricus*,* B. longum*,* B. breve*, and *Streptococcus thermophiles* supplements. Serum insulin and plasma glucose levels significantly decreased as a result of these treatments [69].

Prebiotics are fermented compounds that alter the constitution and/or activity of an individual’s gut microbiota. Prebiotics are composed of polyunsaturated fatty acids (PUFAs), polyphenols, and carbohydrates, which include xylooligosaccharides (XOS), fructans, lactulose, inulin, and fructooligosaccharides (FOS) and galactooligosaccharides (GOS) [70]. Prebiotics were shown in certain studies to promote the growth of Lactobacillus and Bifidobacterium, which results in a significant increase in HDL-C levels and a significant decrease in fasting plasma glucose, serum TG, total cholesterol, and LDL-C levels [52]. According to a study findings, frequent consumption of resistant dextrin, a prebiotic, might aid in the control of metabolic parameters and possibly reduce hyperandrogenism and menstrual cycle irregularities in PCOS patients [64]. Prebiotics improve metabolic markers through modifying the overall composition of microbiota. Studies have demonstrated a substantial correlation between prebiotic treatment and lower rates of appetite and higher concentrations of the gut peptides peptide YY and glucagon-like peptide 1 [71].

Synbiotics are a combination of prebiotics and probiotics. Prominent and productive synbiotics include *Bifidobacterium* and FOS, Lactobacillus and lactitol, and *Bifidobacterium* and GOS. Synbiotics modulate the metabolic profile of PCOS patients by synthesizing SCFAs, functioning as anti-inflammatory agents [52]. Nasri et al. and Samimi et al. found that 12 weeks of synbiotic supplementation with *L. acidophilus*,* L. casei*,* B. bifidum* and inulin improved blood SHBG levels while decreasing HOMA-IR and serum insulin levels in women with PCOS [72]. Further research is necessary to understand the efficacy of various probiotic and prebiotic strains and doses, identify the optimal treatment method, and demonstrate the beneficial impact of probiotics, prebiotics, and synbiotics on PCOS clinical outcomes.

Precision microbiome-based therapies represent personalized treatments for diseases targeting an individual’s gut at the specific microbial community. Using metagenomic profiling, microbiome analysis, and functional genomics, these therapies try to optimize the gut microbiome in ways that may influence metabolic and hormonal processes. In PCOS, with variability in the microbiome composition of individuals, the interventions would be personalized with specific probiotic strains, dietary modification, or FMT to restore microbial balance and treat the metabolic and hormonal disruptions of the condition [73]. Postbiotics, bioactive metabolites of probiotic bacterial fermentation, offer a new therapeutic approach with substantial potential for PCOS management. These include SCFAs, bacteriocins, and other metabolites that are vital in the regulation of gut health, systemic inflammation, and insulin sensitivity, all of which are key elements of PCOS pathophysiology. The SCFAs improve gut barrier integrity, modify immune responses, and control metabolic pathways like glucose homeostasis and lipid metabolism. Additionally, endocrine pathways can be modulated by postbiotics, which reduce androgen levels and improve ovarian function. Promotion of GLP-1 secretion further supports metabolic and hormonal balance. A stable, non-live alternative postbiotic might provide a stable metabolic and immune health in the management of PCOS [74]. Genome-editing tool, CRISPR-Cas9, enables precision in the modification of the microbiome by allowing for the manipulation of specific populations of microbes. With this, scientists can specifically encourage beneficial microbes or suppress pathogens, thus representing a new modality for intervention in the gut microbiota [75].

Research has demonstrated the distinct effects of plant-based and animal-based proteins on the gut microbiota. Specifically, it has been observed that plant-based proteins decrease the numbers of *Bifidobacterium* (B.) *adolescentis* and increase *Bacteroides*, *Alistipes*, and *Bilophila*, consequently elevating the risk of cardiovascular disease. Conversely, studies have shown that plant proteins raise *Bifidobacterium* and *Lactobacillus* while lowering *Bacteroides fragilis* and *Clostridium perfringens*. This has the beneficial effect of increasing levels of SCFAs, which results in reducing inflammation. The authors also found that a high-fat diet increases *Bacteroides* counts and is related to reduced *Lactobacillus* intestinalis along with higher *Clostridiales*, *Bacteroides*, and *Enterobacteriales*, which are associated with inflammation. High concentrations of glucose, fructose, and sucrose have been shown to promote *Bifidobacteria* and decrease *Bacteroides* in association with carbohydrates. Whole grains and wheat bran, which are non-digestible carbohydrates, appear to be related to higher levels of *Lactobacilli* and *Bifidobacteria* [61].

## Conclusion

The relevance of gut microbiota in the pathophysiology of PCOS is highlighted by this review, especially in terms of modulating diabetes, insulin resistance, inflammatory processes, and hormone metabolism. The gastrointestinal tract contains a trillion bacteria, which constitute the gut microbiota. This ecosystem in the gut is essential for reproductive and metabolic health. Dysbiosis, or microbial imbalance, has been reported in PCOS patients, demonstrating a significant correlation between microbial health and PCOS progression. Alterations in the gut microbiota can disturb hormonal balance, increasing hyperandrogenism, which is a characteristic of PCOS. In consequence, increased androgen levels may impact the integrity of the intestinal barrier triggering a vicious cycle of inflammation and hormone disruption. A typical characteristic of PCOS is chronic low-grade inflammation, which exacerbates insulin resistance and metabolic abnormalities. Inflammation and insulin resistance can be sustained by gut dysbiosis by increasing the production of pro-inflammatory cytokines and endotoxins, which can enter the bloodstream through a damaged gut barrier. Reducing inflammation through gut microbiota control offers a possible treatment strategy for PCOS. Insulin resistance, which is key to PCOS, is strongly connected to gut microbiota. In part, the microbiome affects insulin sensitivity and the metabolism of glucose via producing SCFAs. In PCOS, dysbiosis can change the gut-brain axis and decrease the generation of SCFAs, which can impact energy balance and appetite regulation. Restoring healthy gut microbiota may decrease hyperinsulinemia, enhance insulin sensitivity, and alleviate PCOS-related metabolic issues. Novel treatment approaches for managing PCOS can be achieved by focusing on the gut microbiota. Probiotics, prebiotics, symbiotic, and FMT have the potential to modulate gut microbiota while enhancing metabolic and reproductive outcomes in PCOS. Further study is required to maximize the efficacy and safety of these medicines, offering promise for better therapy options and quality of life for women suffering from this complicated condition. While gut dysbiosis has been identified in individuals with PCOS, the evidence is currently suggesting that it could play a contributing role in the pathophysiology of the disorder. Dysbiosis is related to metabolic and hormonal imbalances and may potentially affect the development of PCOS. However, the nature of this relationship remains largely unexplored, and more research is needed to study the alterations in gut microbiota that might influence PCOS and its symptoms.

### Limitations

A significant number of the data referred in this study were based on studies conducted in China and in a few other areas. The limitation on geography might restrict the generalization of these results to other ethnicities and regional populations. Such validation studies in diverse populations should be performed in future research.

## Data Availability

Not applicable.

## Abbreviations

BCAAs: Branched-chain amino acids
CYP17A1: Cytochrome P450c17
DOGMA: Dysbiosis of gut microbiota
FMT: Fecal microbiota transplant
FOS: Fructooligosaccharides
FXR: Farnesoid X receptor
GATA3: GATA binding protein 3
GDCA: Glycodeoxycholic acid
GLP-1: Glucagon like peptide 1
GOS: Galactooligosaccharides
HA: Hyperandrogenism
HDL-C: High density lipoprotein-cholesterol
HFD: High: fat diet
HOMA-IR: Homeostatic model assessment of insulin resistance
HPO: Hypothalamic-Pituitary-Ovary axis
IL6: Interleukin 6
ILC3: Innate lymphoid cell type 3
IR: Insulin resistance
LDL-C: Low density lipoprotein-cholesterol
LH: Luteinizing hormone
LBS: LPS binding protein
LPA: Lipopolysaccharides
MD2: Marrow differentiation 2
NF: kB: Nuclear factor kappa B
PCOM: Polycystic ovary morphology
PCOS: Polycystic ovary syndrome
PNA: Pre-natal androgen
PUFAs: Polyunsaturated fatty acids
PYY: Peptide YY
SCFAs: Short-chain fatty acid
TG: Triglycerides
TGR5: G protein-coupled bile acid receptor
TLR: Toll-like receptors
TUDCA: Tauroursodeoxycholic acid
VLDL: Very low-density lipoprotein
XOS: Xylooligosaccharides
